# THE BALTIMORE LONGITUDINAL STUDY OF AGING: OPPORTUNITIES FOR RESEARCH ON WOMEN'S AGING AND HEALTH

**DOI:** 10.1093/geroni/igz038.1290

**Published:** 2019-11-08

**Authors:** Eleanor M Simonsick, Eleanor Simonsick, Ann Z Moore, Michelle Shardell, Nancy C Shaffer, Toshiko Tanaka

**Affiliations:** 1 Longitudinal Studies Section, Intramural Research Program, National Institute on Aging, Baltimore, Maryland, United States; 2 Intramural Research Program, National Institute on Aging, Baltimore, Maryland, United States; 3 National Institute on Aging, Baltimore, Maryland, United States

## Abstract

The Baltimore Longitudinal Study of Aging (BLSA), an ongoing continuous enrollment cohort study of normative aging established in 1958 currently conducted by the NIA Intramural Research Program, began including women in 1978. To date, nearly 1200 women aged 17-94 at enrollment (median=53 Q1-Q3=40-70) have been followed for up to 21 visits spanning 40 years (median visits=6; Q1-Q3=3-9). Over 3 days, participants receive comprehensive examinations, interviews, imaging and functional and cognitive evaluations; repeat visits occur every 1-4 years depending on age. The BLSA offers opportunities to examine distributions of, change in and interrelationships among several rarely concurrently ascertained parameters (e.g., cardiovascular fitness, resting metabolic rate, glucose challenge response, five-factor personality, brain volumes, and diet) over the life course and across birth cohorts. The BLSA also maintains an extensive biorepository. This talk will summarize the extensive measurement catalogue and timeline and provide illustrative examples from ongoing research on women’s aging and health.

